# Acceptability, Adaptability, and Feasibility of a Novel Computer-Based Virtual Counselor–Delivered Alcohol Intervention: Focus Group and In-depth Interview Study Among Adults With HIV or Tuberculosis in Indian Clinical Settings

**DOI:** 10.2196/35835

**Published:** 2022-05-27

**Authors:** Nishi Suryavanshi, Gauri Dhumal, Samyra R Cox, Shashikala Sangle, Andrea DeLuca, Manjeet Santre, Amita Gupta, Geetanjali Chander, Heidi Hutton

**Affiliations:** 1 Byramjee Jeejeebhoy Government Medical College–Johns Hopkins University Clinical Trials Unit Pune India; 2 Bloomberg School of Public Health Johns Hopkins University Baltimore, MD United States; 3 Department of Medicine Byramjee Jeejeebhoy Government Medical College and Sassoon General Hospital Pune India; 4 Department of Psychiatry Byramjee Jeejeebhoy Government Medical College and Sassoon General Hospital Pune India; 5 School of Medicine Johns Hopkins University Baltimore, MD United States

**Keywords:** computer-based-intervention, HIV/TB clinical setting, alcohol use disorder (AUD), alcohol, India, HIV, TB, feasibility, acceptability

## Abstract

**Background:**

Unhealthy alcohol use is associated with increased morbidity and mortality among persons with HIV and tuberculosis (TB). Computer-based interventions (CBIs) can reduce unhealthy alcohol use, are scalable, and may improve outcomes among patients with HIV or TB.

**Objective:**

We assessed the acceptability, adaptability, and feasibility of a novel CBI for alcohol reduction in HIV and TB clinical settings in Pune, India.

**Methods:**

We conducted 10 in-depth interviews with persons with alcohol use disorder (AUD): TB (6/10), HIV (2/10), or HIV-TB co-infected (1/10) selected using convenience sampling method, no HIV or TB disease (1/10), 1 focus group with members of Alcoholics Anonymous (AA; n=12), and 2 focus groups with health care providers (HCPs) from a tertiary care hospital (n=22). All participants reviewed and provided feedback on a CBI for AUD delivered by a 3D virtual counselor. Qualitative data were analyzed using structured framework analysis.

**Results:**

The majority (9/10) of in-depth interview respondents were male, with median age 42 (IQR 38-45) years. AA focus group participants were all male (12/12), and HCP focus group participants were predominantly female (n=15). Feedback was organized into 3 domains: (1) virtual counselor acceptability, (2) intervention adaptability, and (3) feasibility of the CBI intervention in clinic settings. Overall, in-depth interview participants found the virtual counselor to be acceptable and felt comfortable honestly answering alcohol-related questions. All focus group participants preferred a human virtual counselor to an animal virtual counselor so as to potentially increase CBI engagement. Additionally, interaction with a live human counselor would further enhance the program’s effectiveness by providing more flexible interaction. HCP focus group participants noted the importance of adding information on the effects of alcohol on HIV and TB outcomes because patients were not viewed as appreciating these linkages. For local adaptation, more information on types of alcoholic drinks, additional drinking triggers, motivators, and activities to substitute for drinking alcohol were suggested by all focus group participants. Intervention duration (about 20 minutes) and pace were deemed appropriate. HCPs reported that the CBI provides systematic, standardized counseling. All focus group and in-depth interview participants reported that the CBI could be implemented in Indian clinical settings with assistance from HIV or TB program staff.

**Conclusions:**

With cultural tailoring to patients with HIV and TB in Indian clinical care settings, a virtual counselor–delivered alcohol intervention is acceptable and appears feasible to implement, particularly if coupled with person-delivered counseling.

## Introduction

It is well known that unhealthy alcohol use, encompassing heavy/hazardous use, binge drinking, and alcohol use disorders (AUDs) [1] is associated with increased HIV transmission, decreased use of and adherence to antiretroviral therapy (ART), lower viral suppression, decreased engagement and retention in care [2,3], more rapid HIV disease progression, and mortality [4-6]. Similarly, a recent systematic review and meta-analysis indicated that there is a nearly 3-fold increase in the risk of incident tuberculosis (TB) among people with AUDs [7,8]. Additionally, many studies show a strong association between unhealthy alcohol use and unfavorable TB outcomes, including treatment default, TB relapse, and death [7,9-11].

India accounts for about a quarter of the world’s TB burden [12] and is ranked third in absolute HIV burden [13], with 23.4 lakhs people living with HIV [14]. Unhealthy alcohol use is rapidly increasing in India with more than 50% of those who drink alcohol at unhealthy levels [15]. In India, the prevalence of AUDs among people with HIV and TB has been reported as 12.3% [16] and 24% [17], respectively. Further, although Indian HIV and TB programs mandate alcohol assessments at intake prior to initiation of HIV and TB treatment, effective evidence-based alcohol treatment programs are not routinely implemented in TB and HIV care settings [10].

Brief alcohol interventions (BAIs; 1-4 sessions) are based on cognitive behavioral therapy, the transtheoretical model, or the information, motivation, behavioral skills (IMB) model with motivational interviewing typically used as the style to deliver the intervention [18]. BAIs can reduce alcohol consumption, improve medication adherence, and reduce viral load among people with HIV [19-24]. Despite demonstrated efficacy, however, numerous patient and provider barriers prevent widespread access to and uptake of these interventions [25,26]. These include patient concerns about stigma and confidentiality and the reluctance or inability to access care, as well as lack of provider time or training [26,27]. Computer-based interventions (CBIs) may overcome some of these barriers. They are cost-effective, can reach a large number of people with unhealthy alcohol use, can be delivered with fidelity, and provide confidentiality and convenience [28,29]. Importantly, CBIs appear as effective as person-delivered interventions at short-term (<4 month) follow-up [30].

To date, CBIs have been largely developed and tested in developed countries but not in resource-limited settings [31]. For CBI interventions to be relevant, scalable, and have community-wide impact in resource-limited settings like India, cultural adaptation is required. This includes incorporating the target population’s values, beliefs, language, concepts, and metaphors or key characteristics, while preserving the intervention’s core theoretical components [32,33]. Cultural adaptation also includes the integration of specific contextual factors including feasibility, organizational capacity for adoption, and acceptability to enhance effective implementation [34,35].

In this study, we reviewed a US-developed, evidence-based CBI delivered by a virtual counselor for the reduction of unhealthy alcohol use among people with HIV [36]. Through interviews with adult patients with HIV or TB and focus group discussions with health care providers (HCPs) in government clinical settings in India, we investigated the specific key characteristics of the existing intervention that would need modification for cultural adaptation to yield an acceptable and relevant CBI and the feasibility of CBI implementation.

## Methods

### Study Design

We conducted a qualitative study in Pune, India, consisting of focus group discussions and in-depth interviews with patients, providers, and stakeholders to evaluate the acceptability, adaptability, and feasibility of a CBI for alcohol reduction. Our semistructured interview guide was developed based on our previous work culturally adapting evidence-based interventions [37]. It was designed to assess and make recommendations regarding (1) virtual counselor acceptability, (2) intervention adaptability, and (3) feasibility. We followed the Consolidated Criteria for Reporting Qualitative Research (COREQ) guidelines [38] to report the study.

### Ethics Approval

The Johns Hopkins School of Medicine institutional review board in Baltimore, Maryland, United States (IRB00174744 / CR00026582), and the Byramjee Jeejeebhoy Government Medical College ethics committee in Pune, India, approved this study.

### Computer-Based Brief Alcohol Intervention Delivered by a Virtual Counselor

On one occasion, participants viewed a single session of a US-developed evidence-based CBI designed for people with HIV who consumed alcohol at unhealthy levels [36]. Our CBI used well-established, evidence-based cognitive behavioral techniques, including personalized feedback, pros and cons of drinking, problem solving high-risk situations, and goal setting for reduction or cessation of alcohol use. The software platform was derived from Computer Intervention Authoring Software, an authoring tool to create and enter intervention script into electronic packages featuring a synthetic text-to-speech engine that reads all questions and speaks aloud to the participant (via headphones); synchronous interactivity; natural language reflections; branching logic; and the ability to incorporate specific images, graphs, figures, text, or videos. The flexible and modular platform allows for efficient content modification for a variety of populations [39,40]. Using a motivational interviewing style, a virtual counselor interacts with participants to provide IMB skills that foster alcohol use reduction or abstinence [41,42]. The virtual counselor is a 3D character named Peedy the Parrot that has over 50 specific actions that can be selected to display interest and empathy and increase participant engagement and motivation with the intervention. Although Peedy is an avian character, it appears anthropomorphic through its animations (eye gaze, facial expressions, and body movements) and script (eg, expressive reflections to user answers).

### Recruitment

Study participants were selected using convenience sampling and provided informed consent prior to enrolling. For the in-depth interviews, adults aged 18 years and older with AUD were eligible and were referred to us through ongoing clinic research studies at a government tertiary care hospital in Pune. AUD was defined for males as having an Alcohol Use Disorders Identification Test–Concise (AUDIT-C) score of 4 or more and for females having a score of 3 or more on the AUDIT-C [43]. Between June and November 2018, we approached 15 eligible individuals, and 10 agreed to participate. Those who refused (n=5) were all males and were unable to participate for the required hour to complete the interview. We also conducted 3 focus group discussions: 1 with members of an Alcoholics Anonymous (AA) group (n=12) and 2 with HCPs from a tertiary care hospital (n=21), including counselors, nurses, and clinicians. All HCPs who participated in the study work in same hospital where the study was conducted. Focus groups were conducted in the afternoon when their outpatient department work was over. All in-depth interviews and focus groups were conducted in an easily accessible, private conference room at a hospital in Pune, India.

### Data Collection Procedures and Analysis

Focus groups were moderated by a study co-investigator (NS), who is an Indian female medical anthropologist, and assisted by the study coordinator (GD), who is also an Indian female medical anthropologist, using an interview guide based on topics reviewed in the CBI and adaptation procedures. Focus groups and in-depth interview participants viewed the intervention, which was displayed screen by screen on a white board using a projector. Both the virtual counselor’s speech and the information on each screen was interpreted from English to the local language, Marathi, by GD and NS. After watching each screen, NS and GD obtained participant feedback using the semistructured field guide. On CBI screens where the virtual counselor asked questions with multiple choice answers, participants of HCP focus groups chose the most common option they encountered in their clinical practice, while AA focus group and in-depth interview participants chose the options applicable or suitable to them. We then discussed the feedback provided by the virtual counselor, Peedy, and reviewed the other answer options and feedback. We sought further suggestions and recommendations from the participants.

The main areas of inquiry expected were as follows:

- Participants were asked “How acceptable is this virtual computer-based counselor to reduce drinking?” HCPs were asked “What are the factors in the acceptance or nonacceptance of this computer-based virtual counselor–delivered counseling tool in a clinical setting?”
- Participants were asked about intervention content acceptability and areas of needed adaptation: “What do you like most about this app? Why? What that you did not like? Why?”
- HCPs were asked about the feasibility of implementation factors: “What barriers and facilitators do you expect to uptake and use with HIV/TB patients in clinical settings?”

All in-depth interviews and focus groups were audiorecorded and notes were taken by GD. In-depth interview and focus group texts were then transcribed in the local language and translated into English. A coding guide was prepared based on inductive and deductive codes. All transcripts were coded by GD and reviewed by NS, and data were analyzed in MAXQDA 12 (VERBI GmbH) software using the framework approach [44].

## Results

### Overview

The majority (9/10) of in-depth interview respondents were males with a median age of 41 (IQR 30-56) years. In-depth interview participants included people with TB (6/10), HIV (2/10), HIV/TB co-infection (1/10) and no HIV/TB (1/10; Table 1). Of our 2 HCP focus groups, 1 was conducted with counselors working in HIV and TB research studies who were predominantly female (female to male ratio 12:1). The other focus group was conducted with HIV clinic HCPs and was majority male (female to male ratio 3:6), including 2 counselors, 3 nurses, 2 doctors, 1 pharmacist, and 1 psychologist. Participants of the AA group were all male (12/12) and did not have a TB or HIV diagnosis. Results were organized into 3 domains reflecting the aims of the study: (1) virtual counselor acceptability, (2) intervention adaptability, and (3) feasibility of CBI in clinic settings. Results are illustrated using direct quotations from the respondents.

**Table 1:** Characteristics of the study participants.

Characteristics		In-depth interviews	Focus group discussions		
			Health care providers from HIV clinic	Research counselors	Alcohol Anonymous group members
Participants		10	9	13	12
Age (years), median (IQR)		41 (30-56)	36 (25-64)	38 (24-43)	53 (30-63)
**Gender**					
	Male	9	6	1	12
	Female	1	3	12	0

### Domain 1: Virtual Counselor Acceptability

First, the majority (8/10) of in-depth interview participants found the Peedy the Parrot virtual counselor to be acceptable and felt comfortable honestly answering alcohol-related questions.

Yes, liked it very much. I have not seen anything like this before and nobody explains things to you so well. Nobody makes you sit and speak like this. (female, in-depth interview participant)

Moreover, some respondents stated a preference for a virtual character over a human counselor.

It is better with the bird than [a person such as a] counselor as you can openly discuss with the bird. (male in-depth interview participant)

HCP focus group participants also found the virtual counselor to be acceptable and reported that they would expect patients to trust the information from the virtual counselor, noting the advantage of confidentiality of a virtual counselor–guided discussion.

App is good and patients will be more confident to share information with the app as it doesn’t ask them their name, and they will feel that information will remain confidential. The patient may also trust the information given by Peedy. (female HCP focus group participant)

The predominantly male HCP and AA focus group participants felt that a male virtual counselor with a loud, confident voice would be more effective in facilitating disclosure of alcohol use and alcohol use–related consequences.

So, maybe a male voice or male person to hear them out might help them understand that it’s more nonjudgmental, it’s more, you know, unbiased in a way. (male HCP focus group participant)

If we look at it statistically, then substance use is more prominent among the male gender... So, when they are talking and a female is guiding them it might make them uncomfortable...like you know, make it difficult for them to address certain things. (male AA focus group participant)

By contrast, the predominantly female HCP focus group participants preferred a female virtual counselor because a female in the role of caregiver is more acceptable and patients would feel more connected to a human character than to a bird character.

...a human face especially female instead of a bird as a virtual counselor because patients would feel more connected to a human than a bird. People better link it to female ‘caregiver’ of family and may feel more comfortable. (female research counselor focus group participant)

In-depth interview participants had no gender preference; although they liked the bird as counselor, few preferred a human virtual counselor over an animal/avian virtual counselor.

It is better with both (male/female [virtual] counselor), nothing different. Both are good. (male in-depth interview participant)

Finally, nearly all respondents agreed that it would be important for a live human counselor to be present in the session. Virtual counselors (whether avian/animal or human characters) were noted to have limitations in communication whereas a human counselor could ensure that the information was more persuasive and compelling.

Virtual counselor is less likely to have convincing power. This is because while talking it may not know which point should be stressed upon, it will speak in the same frequency, in the same tone. It will not have feelings and emotions based on the respondent’s responses. (male HCP focus group participant)

Only thing is [virtual counselor] will not have that human feel that they can have with counselor. However, it is good to use it in combination with [a human] counselor. (female research counselor HCP focus group participant)

### Domain 2: Intervention Content Acceptability and Areas of Needed Adaptation

In-depth interview and focus group participants made several recommendations for adaptations to the CBI key characteristics in the areas of drinking triggers, standard drink definition, and IMB skills needed to reduce alcohol consumption. Table 2 shows these recommendations and associated quotations. For local adaptation, participants advised incorporating triggers such as locality and family background, relief from physical pain and stress from family, and type of work. More information was deemed necessary on types of local alcoholic drinks.

Notably, there was a mixed response to the virtual counselor’s discussion of the effect of alcohol on HIV/TB and on gradual versus immediate cessation of drinking. The in-depth interview participants, although diagnosed with HIV or TB, tended not to see the connection between alcohol use and poor health outcomes, whereas AA and HCP focus group participants agreed with the effects and suggested adding more information. HCP and AA focus group participants were concerned that patients tend to have a limited understanding of the impact of alcohol use on HIV and TB drug interactions and on health outcomes such as sexual dysfunction and liver and kidney function. They observed that this knowledge potentially could motivate reduced consumption. Additionally, both in-depth interview and AA focus group participants tended to advocate for abstinence rather than reduction in use of alcohol. Finally, respondents suggested adding behavioral skills to engage participants in (1) religious and spiritual events, (2) learning to refuse alcohol offered by peers, and (3) the willpower to stop drinking alcohol.

Finally, it was suggested that the CBI will be more acceptable if it contains illustrations, especially to aid nonliterate users. Twenty focus group participants suggested that adding more images of major CBI points in addition to the oral presentation by the virtual counselor could improve comprehension and effectiveness.

If [CBI] shows “under what good things can happen if you quit alcohol” that a man after quitting, enjoying with family and having fun with children,” it would have a good impact. (male HCP and AA focus group participant)

**Table 2:** Qualitative data presentation using the information, motivation, and behavioral skills model.

Computer-based intervention domain			Agreement with current content	Data source		Suggestions for adaptation		Quotations
**Information**								
	Reasons for drinking; triggers	All participants agreed with the list of triggers with few adaptations		IDI^a^	Type of occupation or work could be added as the reason for drinking		“I work in a morgue and it is not possible for me not to drink. I daily deal with dead bodies so I used to drink every day. My work is like that.” (male participant)	
				FG^b^: AA^c^ and HCP^d^	Include the influence of the surrounding environment, family background, and locality as major contributions to unhealthy alcohol use, including: Indian culture, drinking alcohol gives confidence, depression due to sexual relationships in HIV discordant couples, alcohol as a pain reliever, feeling of inferiority complex, unemployment, and sometimes to avoid family responsibilities		“Ninety percent of alcoholics are children of alcoholics only. That’s my experience. Dysfunctional family, environmental reasons...environmental reasons mean suppose if a person born in a settlement that has all drunkards around him; so, he inherits that. So, he finds it very common and engage in drinking habit.” (male AA participant) “I used to drink so that no one would ask me to do any work as I am ‘drunk.’ This was an easy excuse to run away from my household responsibilities.” (male AA participant) “If you drink you feel on top of the world and gain confidence to talk and do things you would not otherwise do.” (male HCP participant)	
	Definition of standard drink; safer and risky levels of alcohol use	Very useful information		FG: AA and HCP	Participants suggested the addition of the term Tadi, which is a popular local alcohol drink; its measuring unit is a balloon (1 balloon=250 mL) Linguistic adaptation advised such as use of local terminologies for alcohol like Tadi and daru Participants mentioned that the information about standard drinks mentioned by Peedy is correct in the western context; however, in an Indian context, people may not be aware of the term ‘standard drink’ and the alcohol content in one standard drink		“If they know how much [drink] they take usually and know how much [alcohol/standard drink] there is in one peg [unit], it will benefit them in terms of reducing the quantity of drink.” (female HCP participant)	

	Association with disease	Agreed		IDI	In IDIs, participants were not able to link their disease condition with alcohol use; they reported that alcohol is not related to their disease condition		“...not because of drinking alcohol but because of sharing used glasses [of alcohol], disease [TB^e^] spreads.” (male participant)	
				FG: AA and HCP	All participants mentioned that TB disease is related to alcohol.		“Alcohol leads to TB for sure...” (male AA participant) “Alcohol use will impact their [patients’] adherence to medicines and their visits to the clinic.” (female HCP participant)	
**Motivation**								
	Negative consequences of drinking	Agreed with consequences reported by Peedy		FG: AA	Some negative consequences of alcohol drinking such as health issues and absence at the workplace Some additional consequences reported by them are sexual dysfunction, effect on family relationship, and the loss of confidence		“Absenteeism (absence) from the job or unable to perform well at their job.” (male AA participant) “Sexual inability to perform 90% inability to perform [sexual activity]... Work area; going late, leaving early, taking leaves, again in financial area, taking loans, taking loan from society. And important consequence is, we commonly see that.” (male AA participant)	
				IDI	Effect on kidneys, sadness, fighting, weakness, and lack of concentration One of the IDI participants reported that alcohol drinking has more disadvantages		“There were no benefits [of alcohol use]. Disadvantages were more. Even in the tension, after drinking I used to get more tensed.” (male participant)	
	Reasons for drinking	Agreed with consequences reported by Peedy		IDI	Participant mentioned that he felt relaxed after drinking alcohol		“After drinking alcohol at least for some time, you feel comfortable. You feel relaxed from the stress, like this.” (male participant)	
**Behavioral skills**								
	Cognitive behavioral techniques to reduce/ quit drinking (delay, discuss, and do something else)	All participants agreed with behavioral techniques suggested by Peedy (delay, discuss, and do something else)		FG: AA and IDI	All except one suggested immediate cessation of alcohol Include religious and spiritual therapy		“It is like this if a person drinks two times in a day and drink one bottle at one time then he should have half a bottle, If one drinks three or two times then he should drink only once in a day if he wants to stop drinking. I have stopped drinking like this only [gradually and not suddenly].” (male AA participant) “Not to drink at all is the only answer for that. Even if I start drinking, I drink 3 to 4 bottles [at a time]. I think it is appropriate to stop [instead of reducing it gradually].” (male IDI participant) “You can also tell patient that even after so much of drinking you are alive that means God want you to live and live a better life. He is giving you another chance to live good life. Patient will start believing. Belief in spiritual power is very important.” (male AA participant)	
					FG participants also highlighted that the patient’s willpower along with peer guidance is very important for quitting Some participants also mentioned that the first sip itself is dangerous so it is important to avoid that craving for first sip and try to keep your stomach full of food. As narrated by one of the AA FG participants		“If we become engaged in something else then thoughts about drinking doesn’t come to mind.” (male AA participant) “One suggestion was sharing your drinking limit with friends before drinking. It could help. Your friend should stop you once you complete your drinking limit.” (male AA participant) “I told you that alcohol is nothing but sugar [level in blood]. So, if I was drinking till yesterday and suddenly stopped taking it from today, then my people [peers from AA group] tell me that you should drink electrolyte powder [salt and sugar powder to be mixed in water and taken for treatment of dehydration], eat sweets, as much as you can, so that your glucose level will be maintained [and you do not crave for alcohol] slowly you [alcoholic person] become okay, not only psychologically but also physically. We have seen this.” (male AA participant)	

^a^IDI: in-depth interview.

^b^FG: focus group.

^c^AA: Alcoholics Anonymous.

^d^HCP: health care provider.

^e^TB: tuberculosis.

### Domain 3: Feasibility in Clinic Settings

All participants noted several features of the CBI that would make it feasible for administration in a clinic setting. They reported that intervention duration (about 20 minutes) and amount of information provided were appropriate for use in medical settings. Most in-depth interview participants (8/xx) found the CBI easy to operate. Some in-depth interview participants (2/10) reported that if they were required to use a cellphone instead of the tablet for the intervention, this would be a barrier as some people did not have an Android phone and were not familiar with its operations.

Yes, will help if it is on mobile because even after drinking, everyone is on the mobile. (male in-depth interview participant)

I mean, I cannot operate touch screen mobile. (male in-depth interview participant)

AA focus group participants noted an advantage to the CBI was its capacity for standardized administration, which may not necessarily occur with a person-delivered intervention.

From this [app], the information will be provided systematically and uniformly and the patient doesn’t feel that these people [counselors] are telling their thoughts. Means this is standard and content will be there. (male AA focus group participant)

HCP focus group participants also identified challenges to intervention implementation. Currently with no alcohol treatment services in their clinics, a CBI could result in increased provider workload if providers needed to be physically present to assist while patients are using the software program.

We will give them an appointment. It may affect their work. They cannot take leave to visit the clinic. Our time is 9 AM to 5 PM and they have the same working hours. Sometimes on these lines, [a patient] may drop out. (female HCP focus group participant)

In addition, they noted that patients have limited time in the clinics and often limited reading literacy.

They [the patients] should be able to read options at least [the patient should be literate enough to read the options presented in the CBI]. (female HCP focus group participant)

Finally, other challenges highlighted were availability of internet access in clinics, confidential space for counseling during outpatient hours, and troubleshooting technical problems. However, they also mentioned that such patients may be handled by appointment in the afternoon time in the clinic.

## Discussion

### Principal Findings

Computerized interventions delivered by a virtual counselor can reduce unhealthy alcohol use among people with HIV in the United States [36] and with adaptation may show promise in India among patients with HIV or TB, where unhealthy alcohol use is also prevalent. Using 10 in-depth interviews and 3 focus groups, we analyzed the components of a US-developed CBI. Our study findings suggest that HIV and TB patients found Peedy the Parrot to be acceptable as a virtual counselor. Using a virtual counselor rather than a person was seen as an advantage to foster disclosure of alcohol use and provide confidentiality, a demonstrated advantage of CBIs [45]. Virtual counselors are accepted as counselors if they are sufficiently anthropomorphic [46] and empathic, which Peedy was programmed to be. AA and HCP focus group participants specifically suggested that a human virtual counselor (versus animal/avian counselor) could improve acceptability and efficacy of the intervention. This observation reflects an active question in the human-computer interaction literature as to the relative significance of anthropomorphism and realism of virtual agents in fostering connection between users and content [47]. A related feature of virtual counselor evaluation was its optimal gender. In this study, all participants of the AA group believed that a male virtual counselor could facilitate greater disclosure and comfort because unhealthy alcohol use is predominantly reported among men in India. By contrast, the mostly female HCP focus group participants had a preference for the female gender for the virtual counselor.

### Comparison With Prior Work

Such differing evaluations between the HCP and AA focus groups align with findings of numerous studies showing that people tend to prefer congruence with their virtual counselors [48,49]. Our findings underscore the importance of providing choice in a virtual counselor to increase engagement, interest, and comfort in interacting with intervention content and the importance of examining choice in improving alcohol use outcomes [50,51].

The CBI content was rated highly, showing the generalizability of evidence-based, core behavior change components; however, additional key content was identified for inclusion. All study participants suggested adding drinking triggers (influence of surrounding and locality, type of occupation, avoidance of responsibilities) and adding motivators (peer counseling and willpower). Study participants also recommended that the CBI define a standard drink of alcohol for the local setting. As there is much variation in beverage-specific drink size and types of alcoholic drinks across regions in India, local standardization for alcohol interventions is imperative for this CBI [52].

Importantly, there were areas of discrepancy between the HIV/TB patient, HCP, and AA focus groups. The focus groups wanted more information provided on the well-established link between harmful effects of alcohol on HIV and TB health outcomes, whereas HIV/TB patients rejected the link between alcohol and poor HIV/TB health outcomes.

In cessation versus reduction of alcohol use, HIV/TB patients and AA focus groups argued for immediate cessation for all patients, although it is well established that gradual cessation is the medically recommended approach if there are concerns about alcohol withdrawal [1]. Such discrepancies reflect the larger challenge to present credible and persuasive but often unwelcome health information. Many CBI nuances such as visual design and personalization, unrelated to the quality of the health information, are being explored to influence credibility judgments that subsequently influence acceptance of health information [53,54]. This suggests that further refinement of our CBI may be needed to incorporate this emerging evidence base.

While our study found that CBI delivery in government HIV and TB clinical settings is feasible, most respondents believed that it should be delivered in the presence of a counselor or therapist and not as a stand-alone therapy. This qualitative finding corroborates a recent meta-analysis [30] showing that human-supported interventions were more effective than fully automated interventions in reducing mean weekly alcohol consumption among adults with unhealthy alcohol use. In virtual counselor–based care for mental health, both clinicians and participants preferred digital technology as a complement to, rather than a replacement of, face‐to‐face treatments [30,54].

CBIs seem to offer advantages over person-delivered care; however, future investigation will explore these advantages. High fidelity, for example, is presumed; however, it can be attenuated by user inattention to or misinterpretation of intervention content [55]. Incorporating standardized evaluation of human-computer interaction measures will be essential to ensuring fidelity. A second area of future investigation will be to routinely identify the active mechanisms of behavior change. So far, CBIs have varied in their incorporation of evidence-based theory, which limits ability to identify effective components of interventions [56]. Finally, as the accuracy of transdermal alcohol sensors develops, CBIs can be paired via mobile phone to provide needed on-demand delivery of alcohol treatment [57].

### Limitations

A limitation of the study is that currently the CBI is available only in the English language. This necessitated interpretation of the virtual counselor dialog by the authors in the in-depth interview and focus groups. This may have fostered the perception of many respondents that the CBI (apart from technical concerns) could not be effectively delivered without the physical presence of a live counselor. Another limitation of the CBI is that provisions must be made for limited reading and technical literacy. In the hospital setting, this would mean that a minority of patients would attend CBI on an appointment basis for assistance from staff. This could potentially limit administration, especially during busy clinic hours. It may be that future iterations of this CBI include more visually based intervention material that is even simpler to operate.

### Conclusions

In summary, CBI appears to be acceptable to HCPs and people with HIV and TB and adaptable to an Indian clinical setting. This promising approach to alcohol counseling based on the IMB model is uniform, structured, organized, and provides intervention fidelity. If effective, CBI would result in not only increased access to evidence-based AUD interventions [58], which are currently not available in Indian HIV/TB clinical settings, but also to improved clinical outcomes and quality of life among people with HIV and TB with unhealthy alcohol use.

## Abbreviations

AA: Alcoholics Anonymous
AUD: alcohol use disorder
AUDIT-C: Alcohol Use Disorders Identification Test–Concise
ART: antiretroviral therapy
BAI: brief alcohol intervention
CBI: computer-based intervention
COREQ: Consolidated Criteria for Reporting Qualitative Research
HCP: health care provider
IMB: information, motivation, behavioral skills
TB: tuberculosis
